# Being a Female Gastroenterologist in Turkey

**DOI:** 10.5152/tjg.2024.22612

**Published:** 2024-02-01

**Authors:** Züleyha Akkan Çetinkaya, Özdal Ersoy, Nurdan Tözün

**Affiliations:** 1 Department of Gastroenterology, Memorial Ataşehir Hospital, İstanbul, Turkey; 2 Department of Gastroenterology, Acıbadem Mehmet Ali Aydınlar University School of Medicine, İstanbul, Turkey

**Keywords:** Women, gastroenterologist, gender equality

## Abstract

**Background/Aims:**

Our aim is to examine the representation of woman gastroenterologists in both work and social life in Turkey and to elucidate the difficulties they encounter during their career pathways or while actively practicing their profession.

**Materials and Methods:**

A self-structured survey consisting of 25 questions was sent via email to all 152 female gastroenterologists. Survey results were received from 140 participants.

**Results:**

Sixty percent of the woman gastroenterologists had marriage–career conflicts, and 74% of them stated that they could not manage work–life balance with their children. Among these woman gastroenterologists, 46.6% of them reported that they had faced carrier-related barriers and challenges while applying for an academic rise or expecting a promotion in their job, 58.5% were exposed to gender mobbing, and 35.6% were subjected to verbal or physical violence. On the other hand, woman gastroenterologists are found to be underrepresented in endoscopic interventions where only one-third of the participants perform invasive endoscopic procedures, and the percentage of those who perform advanced endoscopy such as endoscopic submucosal dissection and endoscopic mucosal resection remains even less as 8.9%. The number of women in leadership positions during their careers is found to be low, and only 2 women were selected as the president of the society since 1959, the establishment time of the Turkish Society of Gastroenterology.

**Conclusion:**

More effort is needed to keep a fair gender balance in Turkish gastroenterology society and to increase the women’s representation in therapeutic endoscopy options and also in leadership positions.

**Main Points**

- Many women gastroenterologists must balance the responsibilities of family life which lead to conflicts between career, marriage, and parenting.
- Nearly half face challenges acquiring academic roles and promotions in their part or full-time work and they face verbal and psychological bullying at their workplaces.
- Representation of women in leadership positions and the rate of achieving technically advanced endoscopic interventions are still far less than for their male colleagues.

## Introduction

Expanding the diversity of digestive system diseases, advances in medications, high volume of patients suffering from gastrointestinal diseases, and rapidly developing advanced endoscopic methods have increased the need for digestive system specialists in the last few decades in Turkey. However, the number of doctors actively practicing gastroenterology is not sufficient to meet the demand. This lack of trained gastroenterologists is even a more obvious problem, when considering female gastroenterologists. According to the Turkish Society of Gastroenterology (TSG) data (mentioned below), the representation of female doctors in gastroenterology is also low despite the fact that almost half of our country’s population (49.9%) are women and that there is an increase in the number of female students entering and graduating from medical schools within the recent years in Turkey.^1^ In addition, there are studies showing that female patients prefer female endoscopists especially for screening colonoscopies.^2,3^

In Turkey, women make an important contribution to the workforce in medicine. This started in the second half of the nineteenth century when women began to receive medical education internationally. However, it was only in 1922 that 6 women were admitted to the medical faculty for the first time in Turkey. One of the first graduates, Dr. Müfide Küley, became an internal medicine specialist, pioneered the establishment of gastroenterology in Turkey, and has been the only woman among other woman graduate physicians to pursue an academic career.^4^ According to the data of the Ministry of Health (MoH), the total number of physicians was reported as 171 259 in Turkey in 2020 where the majority of physicians are men (while 37.5% of the physicians are women, male physicians make up 62.5% of the physicians).^5,6^ At the time of our study (October 2019), the official number of gastroenterologists registered with the TSG was reported as 906 with 82 fellows in gastroenterology. As of 2021, only 200 of the 942 gastroenterologists registered with the TSG are women.

Various factors play a role in the selection of female physicians to specialty and subspeciality training in Turkey. While it was rare for women to be admitted to surgical departments before the national medical specialty entry examination was made compulsory for medical graduates to be placed in any specialty training program, an improvement in the balance of gender distribution started after this. In general, since surgical or interventional branches of medicine require more physical strength, the prejudice that men are more suited to these specialties is gradually changing, and more female physicians are applying to these according to their examination results. The diversity of the interventional procedures in gastroenterology, the high income generated, and the visibility of those who choose this field have made gastroenterology a preferred area of specialization for both women and men. The need for female gastroenterologists has increased after the establishment of colon cancer screening programs internationally. However, there are a very few female gastroenterologists compared to men to achieve this. It has always been claimed that gastroenterology is not an attractive field to women due to restrictive reasons such as a challenging work–life balance, marriage, having children, and family duties.

Our aim in this study was to reach women gastroenterologists in Turkey through a questionnaire, to determine their position in both social and working life and the difficulties they face during their advancement of their career, and to create a document that will shed light on the work to be done to close the gender gap in gastroenterology.

## Materials and Methods

The records of the TSG members were accessed via telephone and internet, and 152 female gastroenterologists were reached. An online survey developed by the authors and composed of 25 questions was sent to the physicians as Google forms via email (Supplementary Material 1). The participants first gave their informed consent to use their data, and then all the responses received were evaluated blindly.

## Results

One hundred forty female gastroenterologists responded to the survey. Four questionnaires were sent blank and thus excluded from the evaluation. In total, 136 replies were evaluated. The results obtained in this study are as follows:

### Demographic Findings, Family, and Work–Life Balance

**Figure 1.**
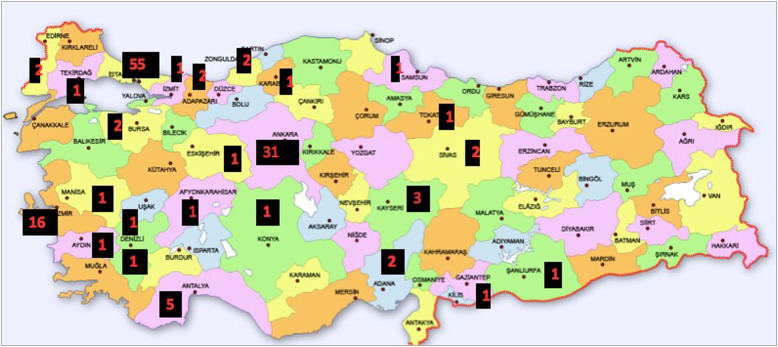
Distribution of female gastroenterologists by provinces in Turkey.

**Figure 2.**
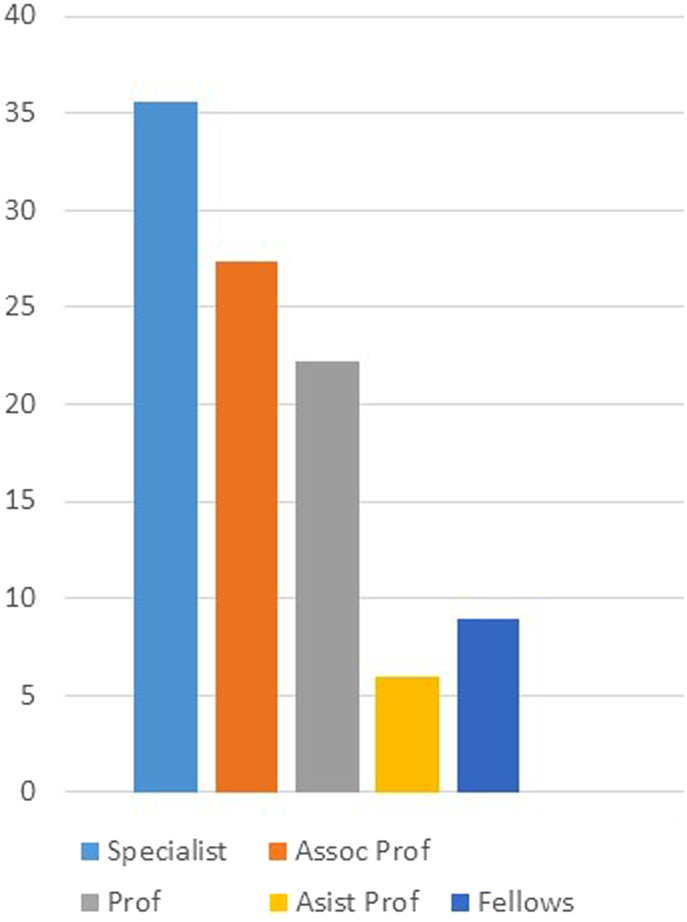
Distribution of female gastroenterologists according to academic position (%).

The majority of female gastroenterologists worked in the most populated cities of Turkey like İstanbul, Ankara, and İzmir (Figure 1). Only 5% of the woman gastroenterologists (WGEs) were divorced where 74.3% were married and the remaining participants were single. Nearly half (44.6%) of WGEs got married between the ages of 25 and 30 while they were practicing internal medicine residentship. The spouses of 64.3% of married WGEs were also physicians. More than half (77.9%) of WGEs had children. Among these, 55 had only 1 child, 40 had 2, 6 had 3, and 1 WGE had 4 children. Sixty percent of the WGEs had marriage–career conflicts, and 73.8% reported that they could not spend enough time with their child/children. Moreover, 40% of the WGEs stated they do the housework by themselves, and only 8.1% of them affirmed that their spouses help them on a regular basis. Ninety-seven percent of the WGEs were actively practicing where only 3% were retired. Percentages of the academic titles of the working WGEs as professors, associated professors, and assistant professors were found to be 22.2%, 27.4%, and 5.9%, respectively. The percentage of gastroenterology specialists having no academical titles was 35.6% of WGEs and that of gastroenterology training fellows was 8.9% (Figure 2).

Forty-five and 14 of non-retired WGEs were working at MoH-affiliated Education and Research Hospitals and State Hospitals, respectively. There were 35 WGEs working at State University hospitals, 14 at Foundation University (private) hospitals, 20 at private hospitals, and 1 in a private office. Some doctors were working at 2 different places.

Considering the working periods of WGEs, 19 of them have been working for 1-5 years, 36 for 5-10 years, 28 for 10-15 years, 26 for 15-20 years, 16 for 20-25 years, 9 for over 25 years, and only 1 female gastroenterologist had been actively working for 36 years.

### Working Issues

Ninety-one percent of the gastroenterologists did not encounter any physical difficulties while performing endoscopy. However, 9% of them complained of numbness in the hands, tendinitis, and leg pain during prolonged endoscopic procedures.

**Figure 3.**
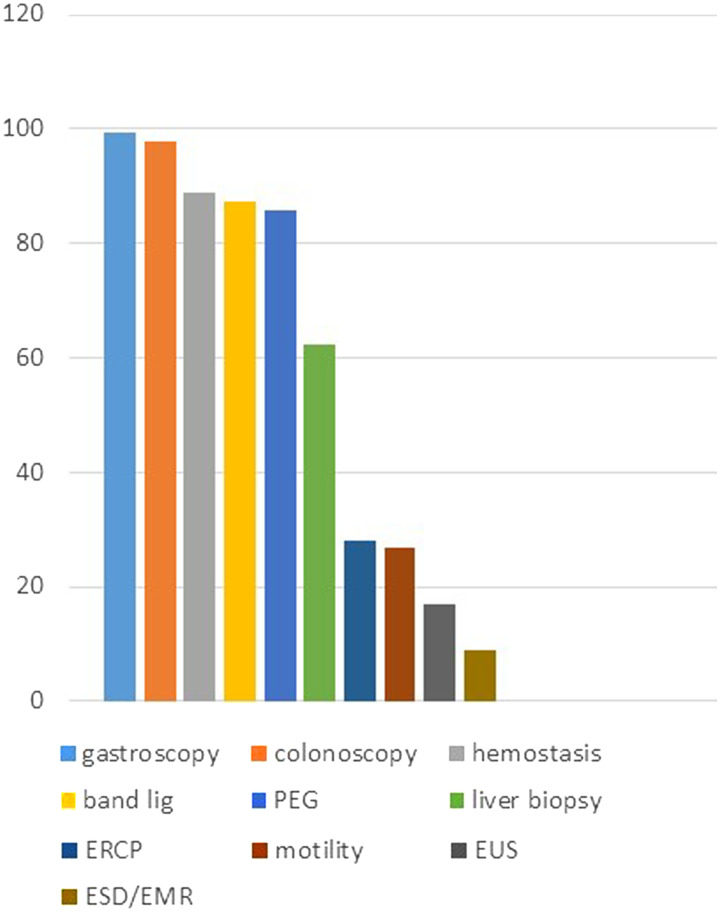
Distribution of invasive procedures performed by women gastroenterologists (%).

Regarding endoscopic skills, the most frequently performed procedures by female gastroenterologists were diagnostic and therapeutic gastroscopy (99.3%) and colonoscopy (97.8%), percutaneous endoscopic gastrostomy (85.9%), band ligation of esophageal varices (87.4%), endoscopic hemostatic interventions (88.9%), and liver biopsy (62.2%). In contrast, advanced endoscopic ultrasound was performed by only 17% of WGEs, endoscopic retrograde cholangiopancreatography (ERCP) by 28.1%, endoscopic submucosal dissection (ESD)/endoscopic mucosal resection (EMR) by 8.9%, and motility tests by 26.7% (Figure 3). More than half of the WGEs (64.4%) did not work on Saturdays. Seventy percent of WGEs did not dedicate time to sports.

Nearly 52% of the physicians had a monthly income/salary of 10 000-20 000 Turkish liras (TRY), 31.1% earned 7000-10 000 TL, 11.1% had more than 20 000 TRY, and 5.9% had a sum of 3000-7000 TRY (United State dollars/TRY exchange rate was at 1/6 in 2019).

### Gender-Related Issues at Work

At the university level, the rate of female physicians who had difficulties in academic promotion due to implicit bias or competition with their male counterparts was 46.6%. About 58.5% of female physicians stated they were exposed to mobbing and 35.6% reported having been subjected to bullying during their career.

Despite the aforementioned challenges, 77% of the female gastroenterologists were satisfied with being a doctor and gastroenterologist and 77.8% of them stated they would choose gastroenterology if they had to decide again.

## Discussion

Internationally, analysis of the workforce by country shows that among some occupations there is a gender differentiation. The number of women is higher in some professions such as education, childcare, nursing, banking, and healthcare, whereas men outnumber women in information technology, surgery, and architecture. The gap widens when considering leadership positions. This may vary depending on the nature of the work as well as cultural, social, and regional differences.

According to the Organisation for Economic Co-operation and Development (OECD) data, the rate of female doctors in Turkey is 40%, which is relatively high compared to other occupational groups.^6^ Considering the distribution of female doctors according to specialties, it is more concentrated in certain branches such as pediatrics, gynecology, dermatology, and basic sciences.^7^ There are no data about WGEs in Turkey in the current literature.

As of 2021, the total number of gastroenterologists registered to the TSG was 942, and only 21% (200) of this number is represented by women. In a society where the male/female ratio is nearly 50/50, it is obvious that this figure is far behind the expectations. But this situation is almost universal since similar data are reported from European and Asian countries. In the United States, 50% of medical school students are women, compared to 36% of gastroenterology residents, the number dropping to 13% at the level of gastroenterology specialists.^8^

Regarding the leadership, the situation is even worse, with relatively few women occupying administrative positions. In this regard, “the critical mass” hypothesis which posits that having 30% of faculty who are women should be sufficient for women to raise their voice and make an observable impact on gender diversity and the sociocultural environment—although challenged by many authors—is still an accepted theory in some communities.^9^ Based on this information, it is evident that there is a gender inequality in some specialties, with gastroenterology being one of them. It has been shown that some specialties are almost entirely composed of male physicians. In 2010, Kuzuca and Arda^10^ examined 28 specialties, and the proportion of male physicians did not fall below 33% in any branch. The highest rate was found to be 66% in the pathology where the rate of female specialists is higher, while this rate is between 79% and 99% in areas where the rate of male specialists is higher. On the other hand, due to natural and culture-related assignment of childcare and homecare to female gender, women choose surgical specialties much less and they prefer branches that have more regular working hours, do not have shifts, and do not prevent them from fulfilling these responsibilities. Although gastroenterology is a subspecialty of internal medicine, it can sometimes be challenging for women physicians due to endoscopic procedures and the high number of emergency cases.

As explained above, one of the most important reasons why women choose gastroenterology less often can be expressed by the fact that “family and household responsibilities are predominantly assigned to women” stemming from the cultural structure. However, the long training period of gastroenterology, the concern of exposure to radiation while in childbearing age, the frequency of night shifts, the high number of emergency cases, the diversity of the invasive procedures, and the absence of part-time work can be listed as the obstacles that women physicians face when choosing gastroenterology. Justifying these arguments, 60% of the women gastroenterologists in our study stated that they had a marriage–career conflict, and 73.8% complained of not being able to spend enough time with their child/children. That is probably why most of the women gastroenterologists in our study opted to have only 1 child.

Gastroenterology is a branch that also requires physical strength. Most endoscopes are not ergonomic for women, and sometimes grasping the buttons and moving the scope is hard. This can cause various physical injuries and conditions such as low back pain, neck and shoulder pain, and tendinitis especially in slender women.^11^ However, in our study, 91% of the WGEs did not experience any problem, and only 9% stated that they had some physical difficulties in endoscopic procedures. In order to overcome this inconvenience, companies that develop endoscopy instruments should manufacture scopes in different sizes which are easier to grasp, more flexible, and less heavy and hence more user friendly for both genders.

A survey study conducted by our authors in 2009 by the Women’s Committee of TSG reported that WGEs performed ERCP less than other endoscopic procedures.^12^ In our survey, we found that nearly one-third (28.1%) of the WGEs performed ERCP, confirming a modest increase in the interest of WGEs in this field. Turkish Society of Gastroenterology’s overseas scholarships and United European Gastroenterology (UEG) fellowships have contributed significantly to this progress. By these initiatives, gastroenterologists of both genders were able to be trained in advanced endoscopy centers abroad (e.g., Japan, Netherlands, USA, and Korea) or clinics working in a specific field (i.e., motility, inflammatory bowel disease, and endoscopic palliation of cancer patients) through bilateral agreements between TSG and these centers. Hands-on courses, applied workshops, or rotations in advanced endoscopy centers have encouraged female physicians to perform invasive procedures over the last few years. This development has shown us that female gastroenterologists can successfully do the same procedures as men simply by an increase in awareness, availability of good mentorship, and provision of equal opportunities for training. As stated previously, in our survey, the fact that a small percentage of female gastroenterologists perform specific endoscopic procedures such as EMR and ESD emphasizes the importance of developing strategies for female gastroenterologists to gain experience in advanced techniques in the future and setting a female quota in advanced endoscopy training courses.

Another important issue is financial income as well as the ability to perform the job. Studies have shown that there are differences in the annual earnings of male and female gastroenterologists, with women doctors earning much less than their male counterparts. When working hours are equalized, women who do the same job earn 22% less than their male colleagues.^13^ In our study, we did not make a comparison of income between male and female physicians, and our women gastroenterologists worked in several different public and private institutions which makes this comparison difficult.

One of the findings of our study is the underrepresentation of women in leadership positions such as head of departments, head of clinic, or presidents of the associations in gastroenterology. Singh et al^14^ also reported that men are clearly in the forefront both in leadership positions and in the management of institutions such as associations, although women take a more active role in academic life than men. In our survey, 55.5% of the physicians had academic titles (e.g., assistant professor, associate professor, and professor). Likewise, the ratio of women at the position of clinical head was found to be significantly lower than overall female physicians working, but there were more females in the position of vice-head which represents a lower rank in Turkish Healthcare administration. This was interpreted as an indicator of the “glass ceiling” phenomenon.^10^ Nearly half of the female physicians who participated in our survey stated that they faced difficulties in academic promotion, and 58.5% reported that they were subjected to bullying. This is a common reality in other professions as well. Cultural norms, acquired behaviors, and conscious or unconscious biases play a role in this issue in male-dominated societies. The low percentage of women in leadership positions can also be explained by the lack of enough role models to give inspiration to their successors in the field. It is noteworthy that the number of female fellows is higher in training programs in which directors or assistant directors are women or in departments managed by women.^15^ Briefly, lack of mentors and also networking among WGEs has a major impact in the underrepresentation of female gastroenterologists in leadership positions.

In line with this, the number of female presidents in gastroenterology associations is less than expected in Turkey compared to abroad. In TSG, the board of directors has changed 17 times since 1986, and only 1 woman has become the president of TSG until 2021. The number of women who became presidents of member societies is slightly higher [4 women in the Inflammatory Bowel Disease Society (IBDS) and only 1 woman in the Turkish Society of Gastrointestinal Endoscopy (TSGE) served as the presidents and a woman president served for 3 terms in the Turkish Association for the Study of Liver (TASL)]. At the time this study was written, a women president has been elected for the second time in the history of TSG followed by the election of women presidents to TASL and the IBDS. In fact, this development gives hope for the future and parallels the increasing awareness about gender equity and equality after the election of women to the presidency of American Gastroenterological Societies (e.g., American Society of Gastrointestinal Endoscopy, American Association for the Study of Liver Disease, American Gastroenterological Association, and American College of Gastroenterology) in 2017 and UEG for the term 2021-2022.

When we look at both anecdotal and published data, it is clearly seen that at least in some societies female patients prefer female gastroenterologists especially for colonoscopic examination.^16^ Cultural differences, religious beliefs, prejudices, or societal characteristics can be put forward as the root of this almost universal preference. In real life, there is worldwide shortage of female gastroenterologists especially for screening colonoscopies. Increasing the number of female physicians in all fields of medicine is important in terms of both providing equal opportunities to men and women in profession and satisfying the unmet needs of the society.

In summary, this is the first study providing information about the working conditions of women gastroenterologists at different institutions in Turkey. The limitation of the study is that male gastroenterologists were not included in the survey, but it is important to note that we were able to reach three-quarters of female gastroenterologists. Despite all the difficulties mentioned above and the fact that gastroenterology is a field that requires physical strength and endurance, and contains a significant role conflict, most of the female gastroenterologists have shown high career satisfaction. Our future goal is to continue this study with a comparison of gastroenterologists from both genders in this context. It is clear that in order to achieve gender equity and equality in gastroenterology, much effort and collaboration between academia, health authorities as decision-makers, and professional societies are needed to fight gender stereotypes and dismantle the existing obstacles on the path to reach this goal. Strategies should be developed to encourage female physicians to choose gastroenterology and to empower female gastroenterologists by providing the same opportunities as males for promotion to leadership positions. There seems to be a long way to go but still achievable when we consider that women may be architects of a better world.

## Supplementary Material 1. Survey Questions

1. Which city do you work in?
2. What is your marital status?
3. Do you have children?
4. If yes, how many?
5. Is your spouse a doctor?
6. How old were you when you got married?
7. What was your title when you got married?
8. What is your current title?
9. Are you still working?
10. What is the type of the institution you work?
11. How many years have you been working in the field of gastroenterology?
12. What invasive procedures are you currently doing?
  - Endoscopic variceal ligation
  - Sclerotherapy and endoscopic hemostasis
  - PEG
  - EMR
  - ESD
  - ERCP
  - EUS
  - Ultrasound-guided liver biopsy
  - Motility test
  - Other
13. Do you encounter any physical difficulties while performing an endoscopy? (inability to grasp the scope’s buttons, etc.)
14. Have you encountered difficulties while applying for an academic career, during your working life, or upon promotions just because of being a woman?
15. Are you working on Saturdays?
16. Have you had a marriage/career conflict throughout your life?
17. Could you/Did you spend enough time with your child/children during your professional life?
18. Who does the housework at home? Does your spouse help you or undertake this job?
19. What is your average monthly income?
20. Do you make time for sports?
21. Are you satisfied of being a doctor?
22. If you had the chance to choose a subspecialty now, would you choose gastroenterology again?
23. If you had the chance to choose a profession now, would you choose medicine again?
24. Have you been exposed to bullying while doing your job?
25. Do you have anything else to add?
